# Experimental Study of Steel–Aluminum Joints Made by RSW with Insert Element and Adhesive Bonding

**DOI:** 10.3390/ma16020864

**Published:** 2023-01-16

**Authors:** Anna Guzanová, Janette Brezinová, Ján Varga, Miroslav Džupon, Marek Vojtko, Erik Janoško, Ján Viňáš, Dagmar Draganovská, Ján Hašuľ

**Affiliations:** 1 Department of Technology, Materials and Computer Supported Production, Faculty of Mechanical Engineering, Technical University of Košice, Mäsiarska 74, 040 01 Košice, Slovakia; 2 Institute of Materials Research, Slovak Academy of Sciences, Watsonova 1935/47, 040 01 Košice, Slovakia

**Keywords:** hybrid joining of dissimilar materials, spot resistance welding, adhesive bonding, load-carrying capacity of joint

## Abstract

This work focuses on joining steel to aluminum alloy using a novel method of joining by resistance spot welding with an insert element based on anticorrosive steel in combination with adhesive bonding. The method aims to reduce the formation of brittle intermetallic compounds by using short welding times and a different chemical composition of the insert element. In the experiment, deep-drawing low-carbon steel, HSLA zinc-coated steel and precipitation-hardened aluminum alloy 6082 T6 were used. Two types of adhesives—one based on rubber and the other based on epoxy resin—were used for adhesive bonding, while the surfaces of the materials joined were treated with a unique adhesion-improving agent based on organosilanes. The surface treatment improved the chemical bonding between the substrate and adhesive. It was proved, that the use of an insert element in combination with adhesive bonding is only relevant for those adhesives that have a load capacity just below the yield strength of the substrates. For bonded joints with higher load capacities, plastic deformation of the substrates occurs, which is unacceptable, and thus, the overall contribution of the insert element to the load capacity of the joint becomes negligible. The results also show that the combination of the resistance spot welding of the insert element and adhesive bonding facilitates the joining process of galvanized and nongalvanized steels with aluminum alloys and suppresses the effect of brittle intermetallic phases by minimizing the joining area and welding time. It is possible to use the synergistic effect of insert element welding and adhesive bonding to achieve increased energy absorption of the joint under stress.

## 1. Introduction

The issue of joining dissimilar materials is an area of interest, particularly in the automotive industry, sustainably focused on a reduction in weight and emissions and performance enhancement. The reason for this is the high degree of automation of the process [1,2]. Joining different types of materials involves two significant factors, namely, welding and mechanical joining, and each of these methods has its own advantages and disadvantages.

Welding involves the different melting temperatures of the materials, resulting in the formation of brittle intermetallic compounds at the steel–Al alloy interface [3,4,5,6,7,8,9,10,11]. Many authors [8,9,10,11,12,13,14] have taken the route of reducing the heat input during welding [3,4,5,6,7,15,16], thereby reducing the thickness of brittle IMCs, or have taken advantage of the better solubilities of Al and Zn compared to the very low solubilities of Fe and Al and have used the zinc interlayer and the associated eutectic reaction for joining [8,9,10,11,12,13]. Experiments were carried out focusing on a reduction in the IMC layer thickness using an ultrasonic-resistance welding process or a novel projection welding technology, consisting of joining an insert element (a short piece of welding filler of suitable composition) to an aluminum plate and then joining the aluminum plate to the steel plate using RSW technology [17,18]. It is also appropriate to support the strength of the joints with, for example, adhesive bonding. Adhesive bonding technology provides many advantages; however, in the bonding process, we encounter the need for surface preparation, which can cause a delay in the production line. For this reason, adhesives requiring no surface preparation or adhesion promoters that are easily applied at room temperature are being developed [19,20]. In the bonding process, it should be kept in mind that the application of adhesives requires curing time, which may cause deformations between Al and steel parts due to different coefficients of thermal expansion. To avoid this undesirable effect, combinations of adhesive and thermal or mechanical bonding are beginning to be applied. Gullino et al. [14] in their experiment showed the advantages of combining spot welding and adhesive bonding using fusion welding and solid-state welding, with a focus on steel–aluminum alloy joining. In addition to the formation of IMCs, another problem arises when joining dissimilar materials with unequal thicknesses, namely, the problem of the subsequent formability of these tailored blanks. The solid-state welding of dissimilar materials (e.g., FSW), compared to fusion welding methods, is more protective of the joint from reducing the formability of the individual materials [21]. Similarly, Aminzadeh in [22] investigated the differences in the formability of joints of unequal-thickness materials formed by laser welding and the TIG method. In practice, however, the automotive industry prefers existing established technologies, such as resistance spot welding (RSW), mainly because of its short welding time, process stability and cost-effectiveness [23].

To achieve a high-quality resistance spot weld in Al steel, some obstructions, such as oxides formed on the surface of the aluminum alloy, must also be dealt with. The oxides formed can result in an increase in the contact resistance between the Al sheet and the welding electrode, thereby limiting the effectiveness of interface heating in the weld area [24,25]. Another problem to be considered in the spot resistance welding of Al on steel is the possibility of electrode wear due to the adhesion of Al to the copper electrode [26]. The author Madhusudana [27] investigated the joining of two materials, 2 mm thick 5054 Al alloy and 1 mm thick galvanized steel, using resistance spot welding. The welds were made with IMC thicknesses of less than 5.5 μm, where the needle-like shape of FeAl_3_ near the Al sheet and the toot-like shape of Fe_2_Al_5_ near the galvanized steel were observed.

When joining hybrid structures, such as aluminum alloy with steel, it is necessary to solve technical problems. Since the properties of the materials to be joined are different, especially the density or melting temperature, during welding, solidification segregation occurs in the weld bath, when inferior joints between aluminum alloy and steel are formed. Residual stresses in the joint occur due to large differences in the coefficient of expansion between the materials being joined, resulting in cracks or the distortion of the structure. The formation of intermetallic compounds and thus probable cracks is also assumed based on the Fe-Al binary phase diagram [28,29]. In some studies, we are informed that the formed Al-Fe IMCs at the interface are an important element to achieve a high-strength joint formed by friction stir welding. In a study of the tensile shear strength of a spot weld of galvanized steel, the value was lower than that of uncoated steel [30,31].

For steel–aluminum joining, mechanical joining methods are widely used due to the low thermal change in the microstructure, as reported in [16,32], and are thus suitable for the bonding process. Joining materials by self-piercing riveting (SPR) is among the most advanced cold-forming methods used for joining aluminum alloys to steel. The advantage is that in mechanical joining, the different melting temperatures of the materials being joined are not a problem. Similarly, brazing and friction stir welding can successfully be used for joining dissimilar materials. Mechanical joining techniques can be very easily combined with adhesive bonding. Ezzine [33,34] successfully focused on hybrid joining using adhesive bonding and riveting. When the requirement is to join dissimilar materials over a larger continuous area, cold-rolling joining comes into consideration. Rahmatabadi [35] tested this in the fabrication of three-layer Al/Mg/Al composites.

The authors were inspired by Zvorykina et al. [18] and designed an experiment focused on joining zinc-coated and deep-drawn uncoated steel to aluminum alloy by spot resistance welding with an insert element and also combined this process with adhesive bonding using rubber- and epoxy-based adhesives. The results obtained could provide more information about joining these materials and the application of the process in the automotive industry.

## 2. Experimental Section

### 2.1. Materials

This experimental research was carried out with the following materials:

- DC04: Deep-drawn, uncoated, cold-rolled low-carbon steel for bodywork, manufactured in compliance with EN 10,130:2006 and EN 10,131:2006. The thickness of the DC04 substrate was 0.8 mm. Hereafter: DC.
- TL 1550-220+Z: Zinc-galvanized fine-grained high-strength low-alloy steel with increased cold formability, manufactured in compliance with VW TL 1550:2008-12 and EN 10143:2006-12. The zinc layer applied was 104 g/m^2^. The thickness of the TL 1550-220+Z substrate was 0.8 mm. Hereafter: TL.
- EN AW-6082 T6: Precipitation-hardened aluminum alloy AlSi1MgMn, manufactured in compliance with EN 573-3, EN-485-1+A1, EN-485-2+A1 and EN-485-4. The substrate thickness of the test specimen was 1 mm. Hereafter: Al.

The mechanical characteristics of the materials used are given in Table 1, and the chemical properties are described in Table 2. The mechanical properties are from a tensile test performed by the manufacturer, and the chemical composition is from the metallurgical certificate provided by the material manufacturer, valid for both the lot and cast number used in experiments.

### 2.2. Shape and Dimensions of Test Samples

**Figure 1:**
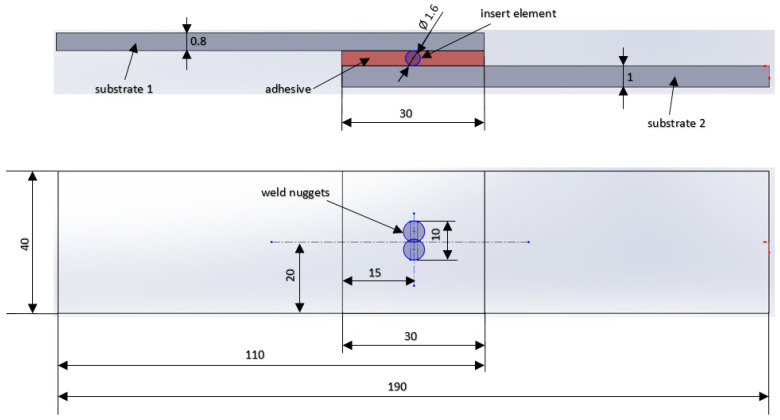
Dimensions of the test joint.

Materials were cut to 40 × 110 mm test samples. Next, the test samples were degreased, coated with an adhesion promoter and joined together by welding, with welding and adhesive bonding having an overlap of 30 mm in the configurations DC-Al and TL-Al (Figure 1). Five test joints were made for each material combination, joining technology and adhesive used (Table 3).

### 2.3. Surface Preparation

The samples were cleaned and degreased in laboratory conditions before joining. Surface preparation consisted of the following immediately sequential steps, applied to both sides of the substrates:

- Degreasing using 5% solution of alkaline degreasing agent (Pragolod 57 N, Pragochema spol. s r. o., Prague, Czech Republic), degreasing time: 10 min; temperature of solution: 60 °C; method: immersion in stirred solution.
- Rinsing in flowing service water, room temperature, 20 s.
- Rinsing in flowing demi water, room temperature, 20 s.
- Application of adhesion promoter based on organosilanes by immersion in solution, immersion time: 10 min.
- Hot-air drying.

The individual surface preparation steps were carried out in five-liter beakers. The individual substrates had a hole made near the edge and were suspended in the solutions on insulated steel wire, 10 pieces per batch. The solutions were sized so that the active ingredients in the solutions were not depleted during preparation.

After this procedure, materials immediately proceeded to adhesive bonding and spot resistance welding.

### 2.4. Microgeometry of the Contact Surface

The contact surfaces of the individual materials were evaluated with a Surftest SJ-301 stylus profilometer in accordance with STN EN ISO 21920-2:2022—Geometric Specification of Products (GPS). The arithmetic mean deviation of the profile Ra, the maximum height of the profile Rz, the mean width of the profile elements RSm and the number of peaks per centimeter RPc were used as monitored parameters. The evaluation length was 4.0 mm, and the λc–profile filter was set to 0.8 mm. Roughness measurements were carried out 10 times on each type of surface, and the arithmetic means of these measurements are presented. The surfaces of the materials were also observed by SEM. EDX planar element analysis was performed in different areas of the joints on a scanning electron microscope EVO MA15 EDX/WDX (Oxford Instruments, Abingdon, UK).

### 2.5. Joining of Materials by Resistance Welding with Insert Element

**Figure 2:**
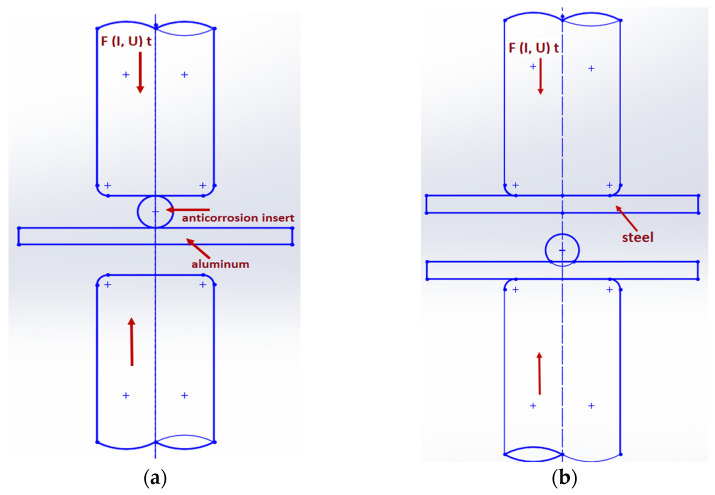
Schematic representation of joint formation by resistance spot welding: (**a**) welding of insert element to aluminum and (**b**) welding of steel plate to insert–aluminum joint.

A novel method of joining by resistance spot welding using an insert element was implemented. In the first stage, the insert element, 308 LSI PR welding wire with a diameter of 1.6 mm and a length of 10 mm, was welded to the Al sample; in the second stage, the welded insert element was covered by a steel plate, and resistance welding was performed again. The joining procedure is shown in Figure 2. Spot resistance welding was carried out on a Nimak Magnetic Drive machine (NIMAK GmbH, Wissen, Germany) with the welding parameters listed in Table 4.

Table 5 shows the chemical properties of the insert element, which is 308 LSI PR welding wire.

### 2.6. Joining of Materials by Resistance Welding with Insert Element and Adhesive Bonding

This type of joint was created by welding the insert element to the aluminum plate. Next, a layer of the adhesive was applied on the aluminum substrate manually using a plastic blade. The thickness of the adhesive was at the level of the insert element height. The joint was then covered by the steel plate, and resistance welding was performed again. As the last step, when double RSW was completed, the curing of the adhesive took place at 175 °C for 25 min.

**Figure 3:**
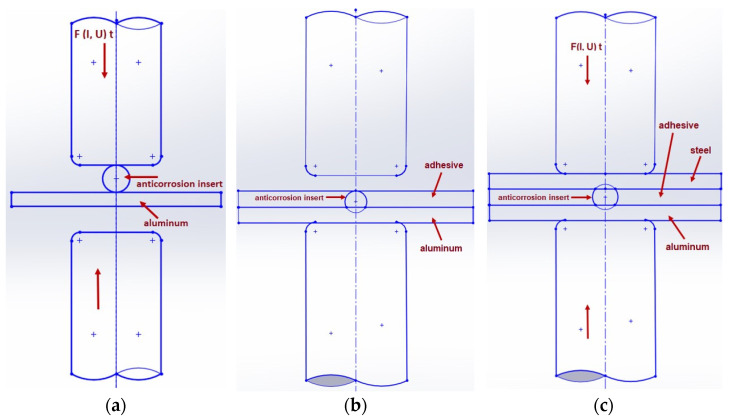
Schematic representation of joint formation using adhesive: (**a**) welding of insert element to aluminum, (**b**) application of adhesive on aluminum substrate, (**c**) welding of steel plate to insert–aluminum joint.

The principle of making joints with an adhesive is shown in Figure 3.

The following types of adhesives were chosen for the experiments, and their properties are described in more detail in Table 6.

- Adhesive 1: TEROSON RB 5197, which is a heat-curing, one-component, rubber-based adhesive with no added solvents.
- Adhesive 2: TEROSON EP 5090, which is a solvent-free, one-component, heat-setting adhesive based on epoxy resins.

Both selected adhesives are used in automotive construction, and both can be combined with other joining technologies, such as spot welding.

### 2.7. Testing of Load-Carrying Capacity of Joints

The load-carrying capacity of the overlapped welded/bonded joints was then tested under tensile stress on a TIRA test 2300 universal testing machine (TIRA GmbH, Schalkau, Germany) at a testing machine ram speed of 10 mm/min, which corresponds to a quasistatic strain rate of 0.0033 s^−1^. After the test, the fracture surfaces of the joints and the type of fracture were evaluated. The quality of the joints was also evaluated by analyzing the contact areas (fracture surfaces) of the joints using the scanning electron microscope specified above.

## 3. Results

### 3.1. Evaluation of Substrate Microgeometry

**Figure 4:**
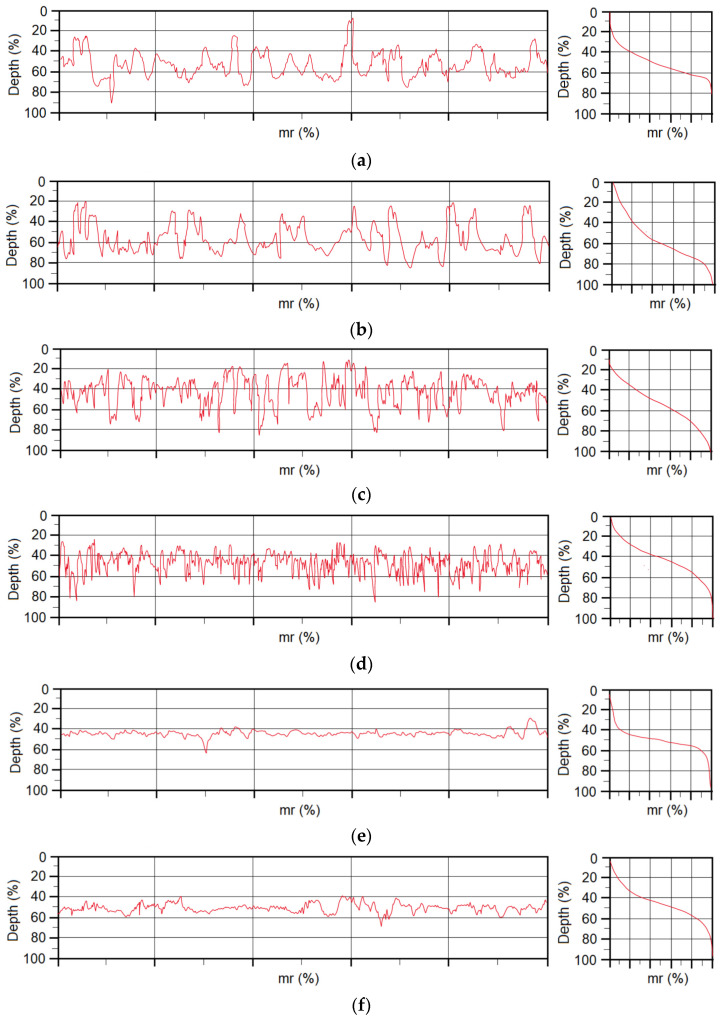
Profilograph of materials in initial state as well as treated with adhesion promoter: (**a**) DC initial surface, (**b**) DC + adhesion promoter, (**c**) TL initial surface, (**d**) TL + adhesion promoter, (**e**) Al initial surface and (**f**) Al + adhesion promoter.

**Figure 5:**
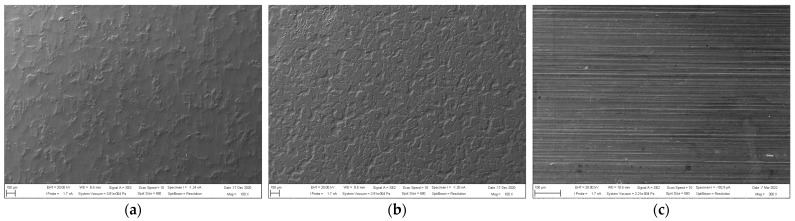
Appearance of materials treated with adhesion promoter: (**a**) DC, (**b**) TL and (**c**) Al.

Based on the methodology described in Section 2.4, the microgeometry parameters of the substrate surfaces were evaluated. The selected parameters of microgeometry are presented in Table 7. Figure 4 shows the surface profiles and the load-bearing profile curves (Abbott curves), and Figure 5 shows the visual appearance of the materials after the application of the adhesion promoter.

From the measured data, it can be concluded that DC and TL steels have very similar vertical roughness parameters (Ra and Rz), while the Al alloy sheet has a significantly lower initial roughness. From the measured values of the horizontal roughness parameters and from the profilographs, it is evident that after the application of the adhesion promoter, the greatest changes in terms of the number of peaks per centimeter occurred on the TL substrate, where the number of peaks increased by 64%; on the DC substrate, it increased by 16%, and on the Al substrate, a decrease in the number of peaks per centimeter of 31% was recorded. The number, height and distribution of peaks and valleys on substrates have a positive effect on adhesion during adhesive bond formation, as they increase the contact area for the formation of chemical bonds to the substrate and, finally, increase the likelihood of the mechanical anchoring of the adhesive as well. For very smooth substrates, it is necessary to either roughen the surface prior to adhesive bonding or to provide adhesion using various adhesion promoters. Organosilane-based adhesion promoters applied on substrates contain molecules able to bond to the metallic substrate on one end and bond to paints or adhesives on the other end. This ‘click chemistry’ can enhance chemical bonding between the substrate and adhesive and also provide resistance against corrosion.

### 3.2. Load-Carrying Capacity of the Joints

#### 3.2.1. Joints Formed by Resistance Spot Welding

**Figure 6:**
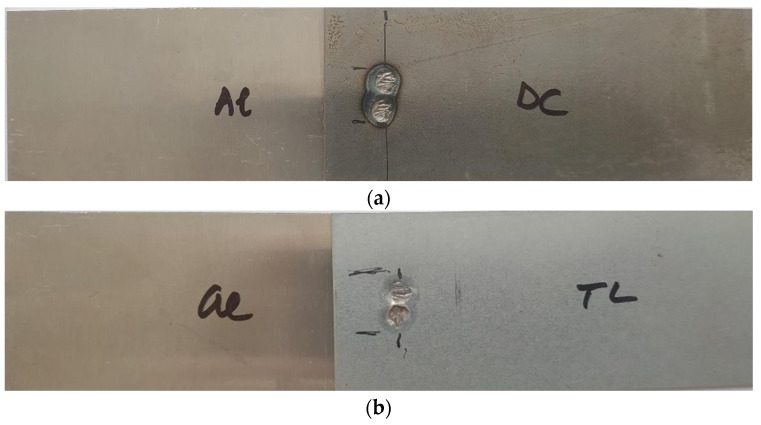
Appearance of test joints made by resistance spot welding: (**a**) Al-DC and (**b**) Al-TL.

Figure 6 shows the appearance of the test joints made by resistance spot welding (Al-DC and Al-TL).

**Figure 7:**
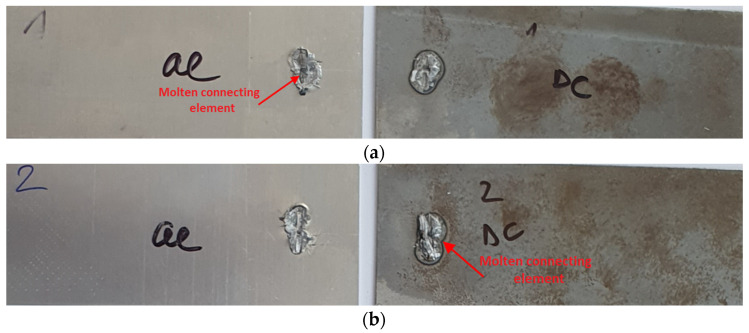
Fracture surfaces of some Al-DC test joints after tensile shear test: (**a**) sample 1 and (**b**) sample 2.

Figure 7 presents representatives of fracture surfaces of Al-DC test joints after the tensile shear test, since the process of joint destruction was the same for all five samples. It can be seen that the molten insert element remained on the aluminum substrate after fracture in four out of five samples (an exception is shown in Figure 7b). The red arrows indicate the locations where the molten insert element remained after destruction. Around the spot weld on the aluminum substrate, the extrusion of the aluminum material from the weld location is visible. Plastic deformation of the samples did not occur in any of the tested joints.

**Figure 8:**
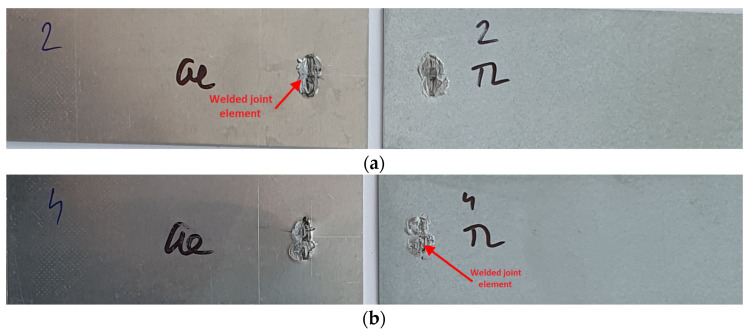
Fracture surfaces of some Al-TL test joints after tensile shear test: (**a**) sample 2 and (**b**) sample 4.

Similar to the Al-DC test samples, only two representative samples are shown after the tensile shear test of Al-TL joints (Figure 8). The same pattern of joint destruction was observed for the four samples, with the greater part of the bonding element remaining welded to the Al substrate, as is shown in Figure 8a. An exception is sample 4 (Figure 8b), where most of the bonding element remained on the TL substrate. On the TL substrate, in the area around the spot weld, extrusion of the Zn layer occurred. As with Al-DC, we do not observe plastic deformation in any sample.

**Figure 9:**
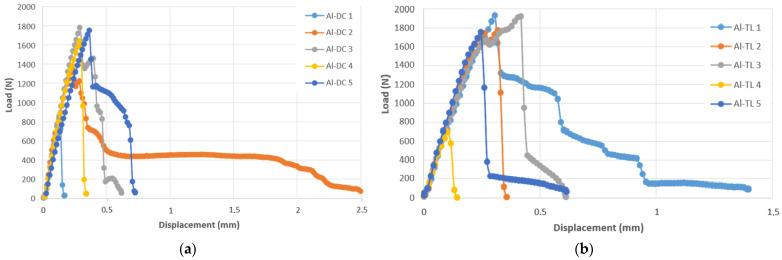
Load–displacement curves for (**a**) Al-DC and (**b**) Al-TL joints.

The load–displacement curves obtained in the tensile shear test of Al-DC and Al-TL test joints are shown in Figure 9. The load curves are nearly linear up to the point of failure and then cascade downward, corresponding to the gradual detachment of the substrates from the insert element. A slightly higher load at failure was recorded for the Al-TL joint compared to the Al-DC joint.

From the maximum load F_max_ at failure and the cross-sectional area A of individual substrates, the stress σ in each substrate was calculated using Formula (1). The aim of this calculation was to prove whether the yield strength (YS) had been overcome in any of the substrates and thus whether plastic deformation in any of the substrates had occurred. The results are listed in Table 8.

(1) $$\sigma =\frac{F_{max}}{A}\;\;\;\;\left[\mathrm{MPa}\right]$$

If we consider the YS of DC steel to be equal to 197 MPa and the width of the substrate is 40 mm with a thickness of 0.8 mm, the load at the yield point equals 6304 N. For the YS of TL steel (292 MPa) and the same dimensions of the substrate, the load at the yield point equals 9344 N, and, finally, for the YS of Al alloy (290 MPa) and sample dimensions of 40 × 1 mm, the load at the yield point equals 11 600 N.

Since the stresses in the substrates (Table 8) are smaller than the yield strength values of Al, DC and TL, no plastic deformation has occurred in any of the substrates.

#### 3.2.2. Joints Formed by Resistance Spot Welding and Rubber-Based Adhesive Bonding

**Figure 10:**
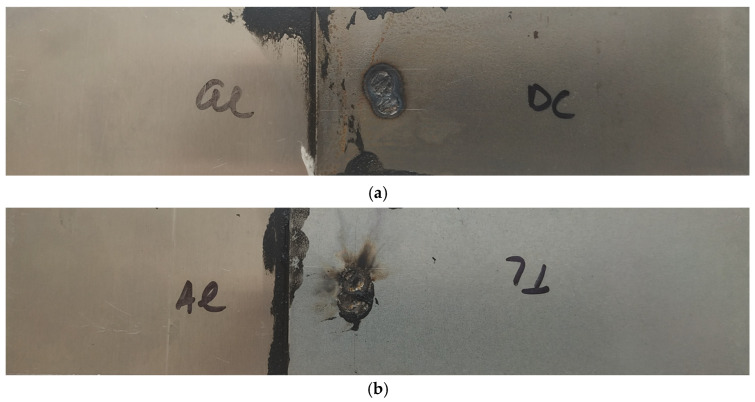
Appearance of test joints made by resistance spot welding and AB: (**a**) Al-DC and (**b**) Al-TL, rubber based adhesive.

Figure 10 shows a test joint made by resistance spot welding with the insert element and adhesive bonding with a rubber-based adhesive (black in color).

**Figure 11:**
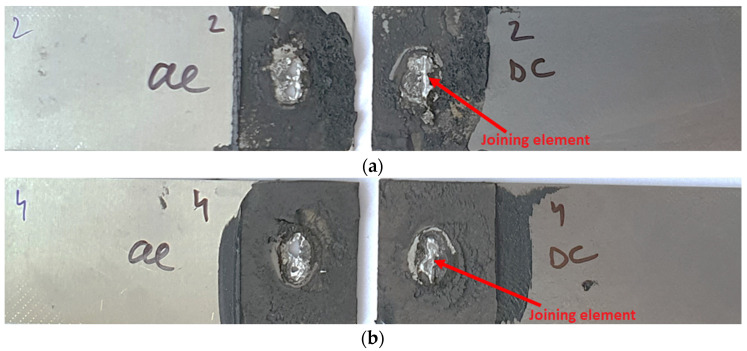
Fracture surfaces of Al-DC test joints made by resistance spot welding and AB after tensile shear test: (**a**) sample 2 and (**b**) sample 4, rubber based adhesive.

Figure 11 presents the test joints after the tensile shear test. The failure process was the same for all samples, so only two samples are documented. The red arrows point to the locations of the insert element, which always remained on the DC substrate. From a macroscopic point of view, no plastic deformation of the substrates is visible. The Al substrate on each of the samples remained perforated after the test, as the joining element, along with a piece of the Al material, was torn away from the Al substrate and remained attached to the DC substrate. This proved the good weld connection between the iron-based insert element and the iron-based DC substrate. However, the bond between the iron-based insert element and Al alloy is also very strong, as the element has been torn out together with part of the Al substrate. The adhesive failure for all samples was 100% cohesive.

**Figure 12:**
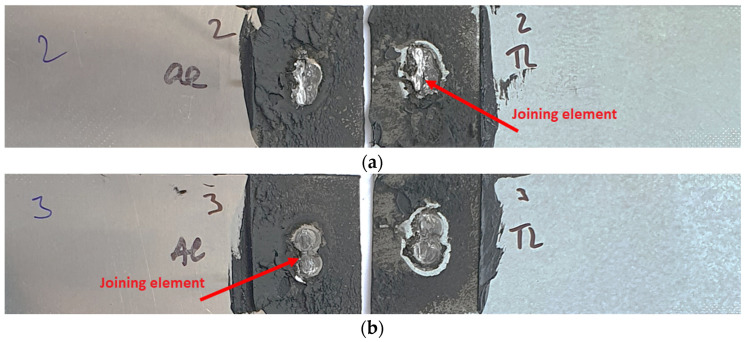
Fracture surfaces of Al-TL test joints made by resistance spot welding and AB after tensile shear test: (**a**) sample 2 and (**b**) sample 3, rubber based adhesive.

The fracture surfaces of Al-TL test joints after the tensile shear test are shown in Figure 12. The red arrows point to the locations where the insert element remained. In this case, due to some, even if limited, solubility of Al and Zn, the insert element remained welded alternately to both the Al (samples 3 and 4) and TL substrates (samples 1, 2 and 5). This means that the joining of the insert element to both surfaces is approximately at the same level. No visible plastic deformation was observed macroscopically. The mode of the adhesive failure is 100% cohesive for all samples.

**Figure 13:**
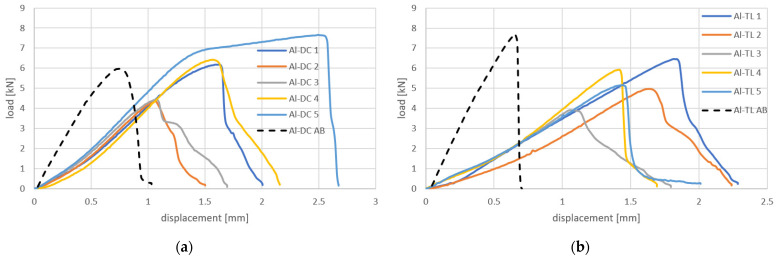
Load–displacement curves for (**a**) Al-DC and (**b**) Al-TL joints made by resistance spot welding + AB, compared with adhesive bonding only (dashed black line).

The load–displacement curves obtained in the tensile shear test of Al-DC and Al-TL test joints are shown in Figure 13. It can be concluded that, compared to joints made by resistance spot welding only, a higher load was necessary to break the joints due to the adhesive bonding contribution. Until failure, the character of the process is nearly linear. For specimen 5 of Al-DC joints, the load exceeded the yield strength of one of the substrates and caused it to strengthen. The load–displacement relationship resembles a stress–strain diagram of the substrate. At some point in the strengthening, the load exceeded the cohesion of the adhesive, and the failure of the joint occurred. The load at the failure of Al-TL joints is similar to that of Al-DC joints with the rubber adhesive, and it varies from 5 to 6.5 kN.

The dashed black lines in Figure 13 indicate the load–displacement curves of adhesive joints only (without welding or the insert element). It can be seen that using hybrid joining technology–resistance spot welding with the insert element in combination with adhesive bonding is manifested by a larger area under the load–displacement curve, i.e., an increase in joint energy absorption is proven.

**Figure 14:**
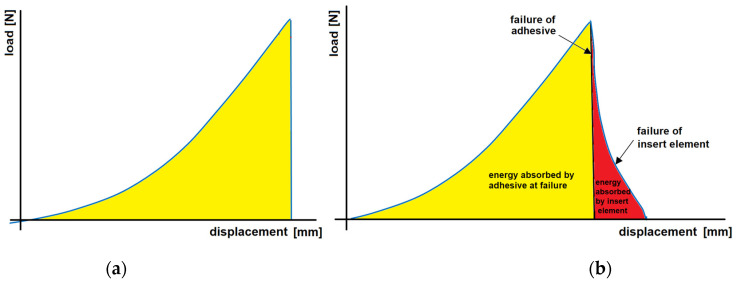
Load–displacement curve for (**a**) pure adhesive-bonded joint and (**b**) hybrid welded joint combined with adhesive bonding.

A typical load–displacement curve of a purely bonded joint has a typical triangular shape, where the load increases linearly with displacement up to the failure of the joint. After a joint failure, the force immediately decreases almost perpendicular to the x-axis (Figure 14a). When using an insert element, a gradual decrease in force from Fmax to 0 N can be seen in the load–displacement curves, achieved under quasistatic loading. This gradual disappearance of force is caused by the insert element. The failure process can best be seen from the comparison shown in Figure 14b.

In Figure 14b, on the descending portion of the curve, the adhesive failure region and the insert element failure region are clearly distinguishable. If we calculate the area under the curve, we can quantify the contribution of the adhesive and insert element to the energy absorbed by the joint. Hence, the importance of using an insert element is in increasing the area under the load–displacement curve and hence not increasing the load capacity of the connection but the energy absorbed by the connection, which is of importance in a crash event.

Table 9 shows the level of stresses in the substrates, from which it can be determined whether the yield strength was overcome and therefore plastic deformation in any of the substrates occurred.

From the above stresses, as well as from the previous load–displacement curves, it is clear that plastic deformation occurred in the DC substrate and in the Al-DC joint (joints 4 and 5) formed by resistance spot welding and adhesion bonding with the rubber-based adhesive.

#### 3.2.3. Joints Formed by Resistance Spot Welding and Epoxy-Based Adhesive Bonding

**Figure 15:**
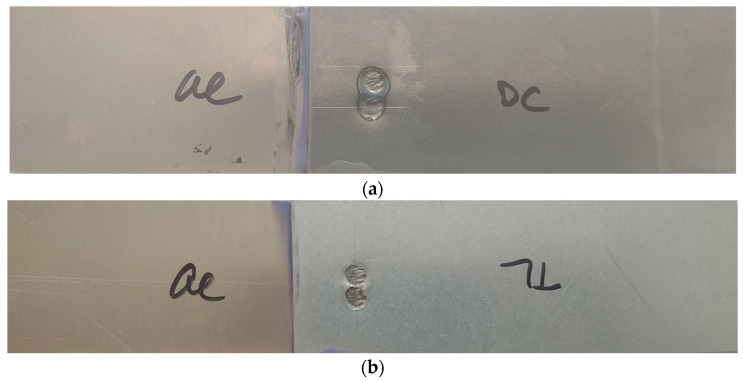
Appearance of test joints made by resistance spot welding and AB: (**a**) Al-DC and (**b**) Al-TL, epoxy based adhesive.

Figure 15 shows test joints made by resistance spot welding with the insert element and adhesive bonding with an epoxy-based adhesive (purple in color).

**Figure 16:**
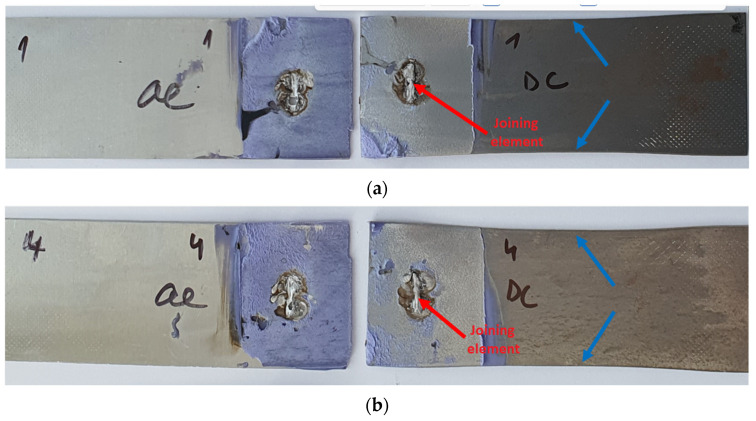
Fracture surfaces of Al-DC test joints made by resistance spot welding and AB after tensile shear test: (**a**) sample 1 and (**b**) sample 4, epoxy based adhesive.

Figure 16 presents Al-DC joints after the tensile shear test. The failure process was the same for all samples, so only two samples are documented. The red arrows point to the locations of the insert element, which always remained on the DC substrate. From a macroscopic point of view, the plastic deformation of DC substrates is visible. Blue arrows indicate the contraction of the DC substrate. The Al substrate on each of the samples remained perforated after the test, as the joining element, along with a piece of the Al material, was torn away from the Al substrate and remained attached to the DC substrate. This proved the good weld connection between the iron-based insert element and iron-based DC substrate. However, the bond between the iron-based insert element and Al alloy is also very strong, as the element has been torn out together with part of the Al substrate. An adhesive–cohesive failure of the bonded area for all samples was identified, but the adhesive failure appears to be dominant over approximately 80% of the bonded area. The adhesive was separated from the DC substrate, always remaining on the Al substrate.

**Figure 17:**
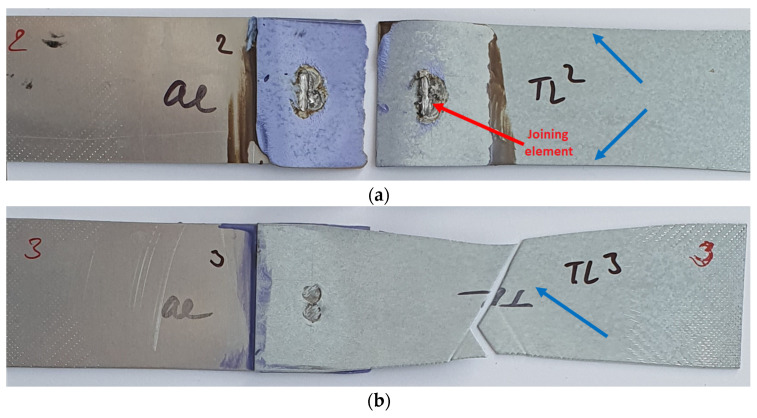
Fracture surfaces of Al-TL test joints made by resistance spot welding and AB after tensile shear test: (**a**) sample 2 and (**b**) sample 3, epoxy based adhesive.

Selected fracture surfaces of Al-TL test joints after the tensile shear test are shown in Figure 17. In this case, the insert element always remained welded to the TL substrate. In some Al-TL joints, the insert element detached from the Al substrate without disrupting it, while for other Al-TL joints, a part of the Al substrate along with the insert was torn off during the loading of the joint, causing Al substrate perforation and leaving the insert element attached to the TL substrate. The exception is joint 3, where the joint remained intact, but the TL substrate broke outside the joint, which means that the joint strength exceeds the tensile strength of the TL substrate. Macroscopic plastic deformation was observed in the TL substrate in all cases. The adhesive failure was mixed adhesive–cohesive for all samples, with the adhesive mode prevailing in approximately 90% of the bonded area.

**Figure 18:**
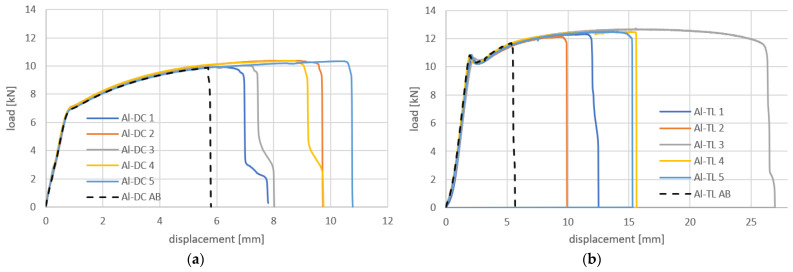
Load–displacement curves for (**a**) Al-DC and (**b**) Al-TL joints made by resistance spot welding + AB, compared with adhesive bonding only (black dashed line).

The load–displacement curves obtained in the tensile shear test of Al-DC test joints are shown in Figure 18a. It can be concluded that, compared to Al-DC joints with a rubber-based adhesive, a higher load was necessary to break the joints due to the adhesive bonding contribution. The loading curves indicate that the load-carrying capacity of the joint is high, and the joint resisted in the region of the elastic deformation of the substrates, as well as in the region of the significant plastic deformation of the substrates. The failure of the connection occurred just before the maximum force was reached in the stress–strain diagram. Thus, it can be concluded that the bearing capacity of the connections is almost at the level of the maximum force that the DC substrate can withstand. At some point in substrate strengthening, the load exceeded the adhesion of the adhesive to the DC substrate, and the failure of the joint occurred at different displacement values.

A graphical representation of the load–displacement curves of Al-TL joints is shown in Figure 18b. In this case, the highest loads required to fracture the joints were applied, compared to Al-DC joints with the epoxy-based adhesive, as well as Al-TL joints with the rubber-based adhesive. The load–displacement curves are identical to the stress–strain diagram of TL steel, with a clearly recognizable yield phenomenon in TL steel. The failure of the joint occurred near the maximum force corresponding to TL steel, but at different displacement values.

Figure 18 also shows that the contribution of the insert element to the total energy absorption during joint loading (deflection on the downward portions of the curves) is negligible compared to the large total area under the loading curve.

Again, dashed black lines in Figure 18 indicate the load–displacement curves of the adhesive joint only (without welding and the insert element). Compared to the pure adhesive joint, a significant increase in the energy absorption of the joint made by hybrid joining was again shown.

Table 10 shows the values of stresses in individual substrates, calculated from the load at failure. In Al-DC joints with an epoxy adhesive, the plastic deformation of the DC substrate only was proved in all tested joints, while in Al-TL joints with the epoxy adhesive, plastic deformation was proved for both the Tl and Al substrates. Macroscopic deformation and failure in the TL-Al pair occurred primarily in the TL substrate due to the higher stress values indicated in Table 10. The stresses in the Al substrate in the Al-TL joint are just above the yield strength (YS_Al_ = 290 MPa), and macroscopic deformation cannot be recognized yet.

#### 3.2.4. Overall Evaluation of the Load-Carrying Capacity of the Joints

Based on the comparison of the individual loads for the different joint types, it can be concluded that the joints formed with the rubber-based adhesive displayed higher values than the joints where no adhesive was used, but even higher values were observed for the joints bonded with the epoxy-resin-based adhesive.

**Figure 19:**
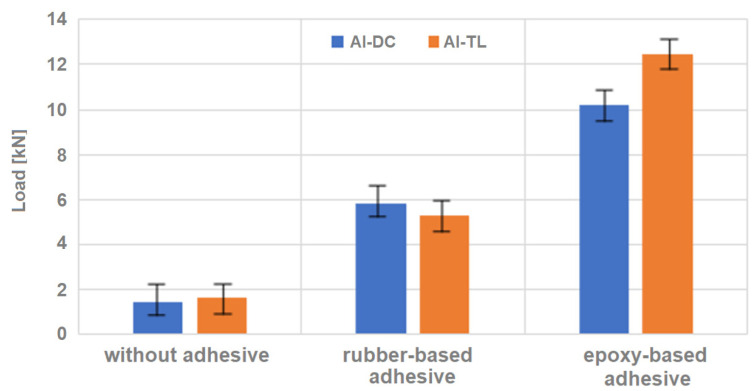
Tensile shear test results of Al-DC and AL-TL samples without adhesive and with two types of adhesives.

Figure 19 shows the average loads at failure for Al-DC and AL-TL joints without an adhesive and with rubber-based and epoxy-based adhesives.

The rubber-based adhesive showed better adhesion to all three types of substrates, where 100% cohesive failure was always observed, and it seems to be suitable to combine this adhesive with insert element welding, because the contribution of the insert element to the overall load-bearing capacity of the test joint is more significant compared to the epoxy-based adhesive. The epoxy-based adhesive gives too much strength, and it is pointless for the strength of the bonded joint to exceed the yield strength of the substrate.

## 4. SEM analysis of Joints

Due to the large difference in the melting points of the insert element and the Al alloy, lower welding parameters were chosen in the first stage of joining. Actually, one cannot even speak of true welding. During welding in the first stage, the Al alloy melted, while the insert element remained unmolten. The liquid Al alloy wets and spreads on the solid steel surface, which creates a special brazed joint (Table 11, left). The insert element has been pressed into the molten Al sheet, and the molten Al alloy has splashed out into the surrounding area (see Table 11, pictures in the center and right). In addition to the metallurgical joint, the insert element is mechanically wedged into melted and solidified Al alloy.

### 4.1. SEM Analysis of Al-DC Connection

**Figure 20:**
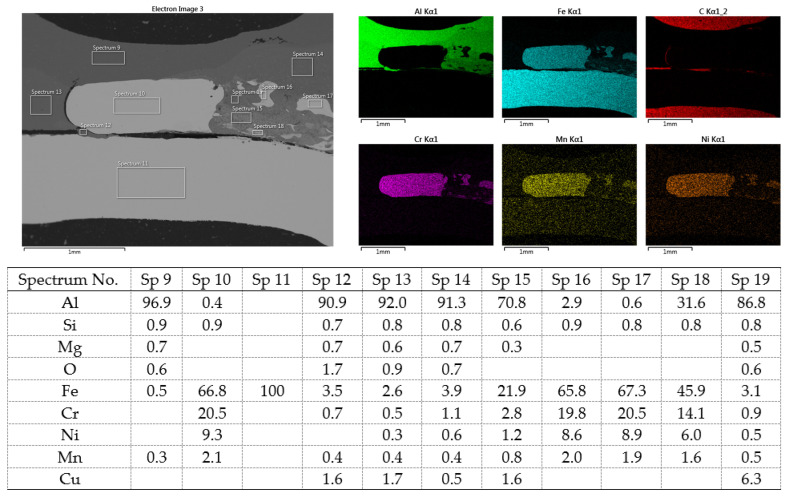
SEM analysis of Al-DC connection, distribution element maps and EDX spectra.

Figure 20 shows the distribution maps of the individual elements on the cross-section of the joint, as well as the results of EDX analysis at selected locations.

**Figure 21:**
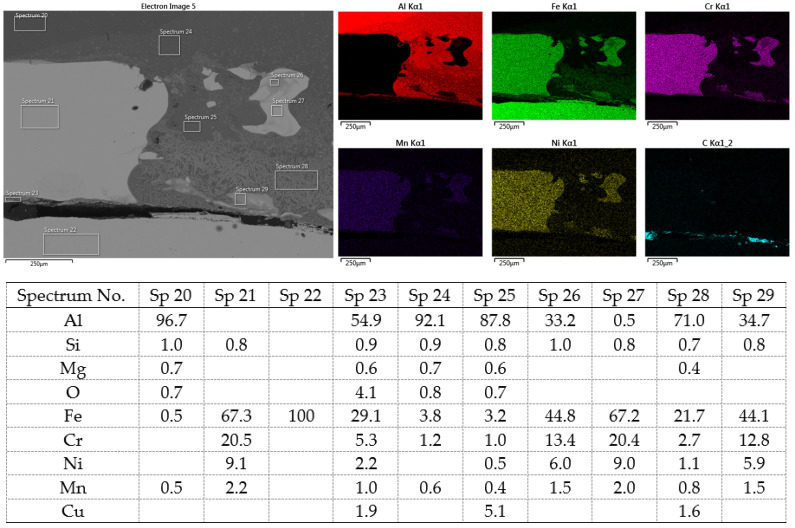
SEM analysis of Al-DC connection, distribution element maps and EDX spectra-detail.

The metallographic section of the intact joint showed the insert element (spectra 10, 16 and 17) sealed in the Al sheet. Spectrum 9 confirms the basic chemical composition of the Al sheet, and spectrum 11 confirms the basic chemistry of deep-drawn low-carbon DC steel. The other spectra span different phases at the Fe-Al interface, ranging from the more aluminum-rich phases located farther away from the steel (spectra 13 and 14) to the more iron-rich phases lying close to the steel (spectra 15 and 18). The SEM analysis of the joint detail at the DC-Al–insert-element interface (Figure 21) more clearly shows this gradient change in Al and Fe contents at the joint interface (see Al and Fe elemental distribution map).

At the interface between DC and the Al melt, regions with a needle-like structure are present (spectrum 28), which, according to [36,37], could correspond to Fe_2_Al_5_ or other phases, e.g., FeAl_3_, FeAl_2_, FeAl or Fe_4_Al_13_. From the point of view of bond strength, it is desirable that there are no IMCs at the Fe-Al interface, or that they are as thin and discontinuous as possible.

### 4.2. SEM Analysis of Al-TL Connection

**Figure 22:**
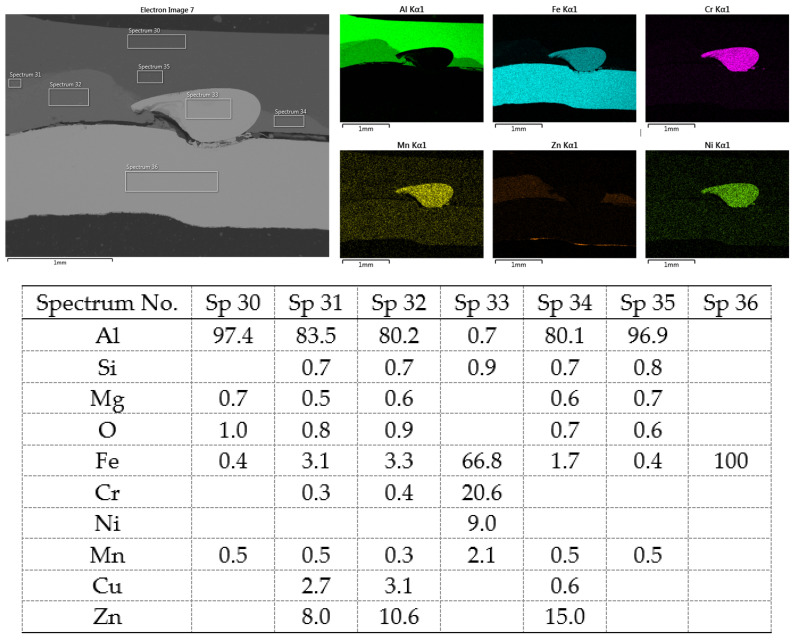
SEM analysis of Al-TL connection, distribution element maps and EDX spectra.

A simpler interface structure without needle-like formations can be observed when joining Al to galvanized HSLA steel (Figure 22).

**Figure 23:**
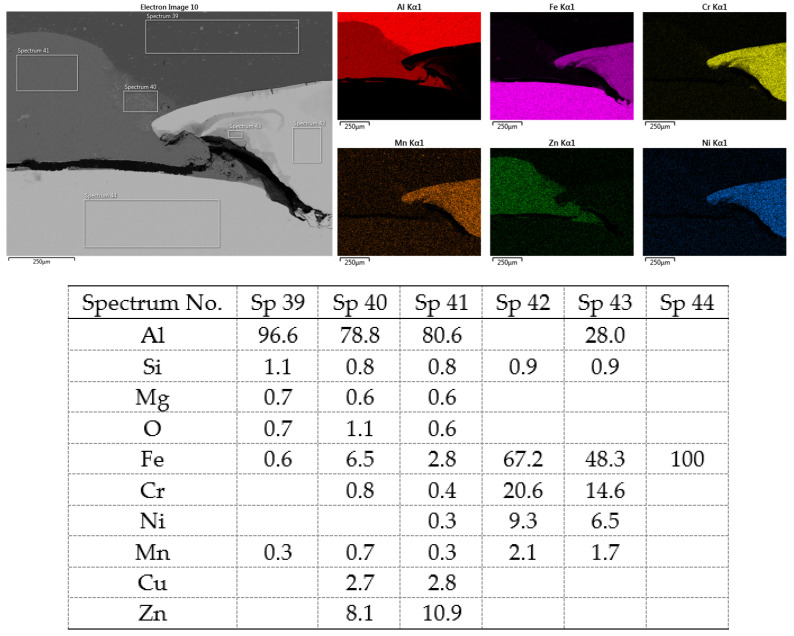
SEM analysis of Al-TL connection, distribution element maps and EDX spectra-detail.

Again, the insert element is pressed into the molten Al alloy during welding. Changes in the structure of the steel are not visible, as no melting has occurred, or if it has, the weld nugget is usually in the core of the steel. However, a relatively thick layer of Al-Zn alloy (spectra 31, 32 and 34)—the Al-rich α phase—is evident in the Al substrate toward the steel–Al alloy interface. It can be seen in more detail in Figure 23, where the distribution map of Zn and Al is particularly interesting, documenting its dissolution in Al.

## 5. Conclusions

The aim of this research was to verify a new method for joining dissimilar materials—resistance spot welding using a joining element and adhesive bonding.

From the results, the following conclusions can be drawn:

- The surface preparation of steels and aluminum alloys with an organosilane-based adhesion promoter led to the excellent adhesion of both adhesives to all substrates, reflected in the high load-bearing capacity of connections.
- Due to the small contact area, connections formed by resistance welding with the insert element alone achieved a load-bearing capacity of up to 1800 N, irrespective of whether they were Al-DC or Al-TL joints. The joint formed is actually a brazed joint, where the molten Al alloy just wets the surface of the insert element and steel plate.
- Joints formed by spot resistance welding and a rubber-based adhesive had a load capacity of approximately 6 kN, while joints with an epoxy-based adhesive had a load capacity at the level of the maximum load capacity of the substrate (Al-DC: 10 kN; Al-TL: 12 kN)
- The importance of the insert element lies in the fact that it increases the energy absorption of the joint during breakage. This is manifested by a change in the slope of the downward part of the load–displacement curve.
- Although the load-carrying capacity of bonded joints and joints formed by hybrid joining technology (RSW+AB) is approximately the same, the use of an insert element causes a significant increase in the energy absorbed by the joint under stress.
- SEM analysis confirmed a strong bond between the insert element and both substrates during load testing. The element remained largely welded to the steel substrate, pulling out a portion of the volume from the Al substrate, which remained firmly attached to the element.
- The spot welding process is fast, which blocks the formation process of IMCs.
- However, the use of an insert element in combination with adhesive bonding is only relevant for those adhesives that have a load capacity just below the yield strength of the substrates. For bonded joints with higher load capacities, the plastic deformation of the substrates occurs, which is unacceptable, and thus, the overall contribution of the insert element to the load capacity of the joint becomes negligible.
- The results of the presented research show that the combination of the resistance spot welding of an insert element and adhesive bonding facilitates the joining process of galvanized and nongalvanized steels with aluminum alloys and suppresses the effect of brittle intermetallic phases by minimizing the joining area and welding time. It is possible to use the synergistic effect of insert element welding and adhesive bonding to achieve increased energy absorption of the joint under stress.

## Figures and Tables

**Table 1:** Mechanical properties and some specific conditions of materials.

	YS [MPa]	UTS [MPa]	Elongation [%]	Thickness [mm]	Conditions
DC	197	327	39	0.8	Electrostatically oiled
TL	292	373	34	0.8	Zn-coated
Al	290	340	14	1.0	Solution-treated, artificially aged

**Table 2:** Chemical composition of materials, wt. %.

**DC**									
C	Mn	P	S	Fe					
0.040	0.250	0.009	0.008	bal.					
**TL**									
C	Mn	Si	P	S	Al	Nb	Ti	Cu	Fe
0.100	1.000	0.500	0.080	0.030	0.015	0.100	0.150	0.200	bal.
**Al**									
Si	Fe	Cu	Mn	Mg	Cr	Zn	Ti	Al	
1.00	0.40	0.06	0.44	0.70	0.02	0.08	0.03	bal.	

**Table 3:** Number of joints made by resistance spot welding (RSW) and hybrid RSW and adhesive bonding (AB) technology.

	RSW	RSW + Adhesive 1	RSW + Adhesive 2
DC-Al	5	5	5
TL-Al	5	5	5

**Table 4:** Spot resistance welding parameters.

	Stage 1	Stage 2
Welding force, F [kN]	2	4
Welding time, t [ms]	10	16
Welding power, I [kA]	8	12

**Table 5:** Chemical composition of insert element, wt. %.

	C	Mn	Si	Cr	Ni	Mo	Cu	Fe
ER 308LSi	0.02	1.8	0.85	20	10	0.2	0.2	bal.

**Table 6:** Selected properties of adhesives, given by adhesive producer (Henkel AG & Co., KGaA, Düsseldorf, Germany).

	TEROSON RB 5197	TEROSON EP 5090
E-module	880 MPa	2 GPa
Tensile strength	12 MPa	35 MPa
Elongation at break	10%	10%
Poisson’s ratio	0.4	0.4
Shear strength (DIN EN 1465)	at 20 °C >15 MPa	>30 MPa
Layer thickness	0.2 mm	0.2 mm

**Table 7:** Selected parameters of surface microgeometry.

	Ra [µm]	Rz [µm]	RSm [µm]	RPc [-/cm]
DC initial surface	0.87	5.12	300.30	34.20
DC + adhesion promoter	0.94	4.66	256.80	39.86
TL initial surface	1.00	5.11	137.8	73.76
TL + adhesion promoter	0.62	4.03	83.0	120.94
Al initial surface	0.15	1.04	142	74.00
Al + adhesion promoter	0.24	1.61	202	51.00

**Table 8:** Maximum load Fmax [N] and stress σ [MPa] in substrates.

		Al-DC			Al-TL	
Sample No.	Fmax (Al-DC)	σ (DC)	σ (Al)	Fmax (Al-TL)	σ (TL)	σ (Al)
1	848	26.52	21.22	1933	60.42	48.33
2	1351	42.22	33.77	1778	55.56	44.44
3	1785	55.78	44.62	1923	60.10	48.08
4	1645	51.39	41.11	700	21.86	17.48
5	1755	54.84	43.87	1758	54.94	43.95

**Table 9:** Maximum load Fmax [N] and stress σ [MPa] in substrates used in welded-bonded joints (rubber-based adhesive).

		Al-DC + AB			Al-TL + AB	
Sample No.	Fmax (Al-DC)	σ (DC)	σ (Al)	Fmax (Al-TL)	σ (TL)	σ (Al)
1	6180	193.15	154.52	6462	201.95	161.56
2	4356	136.11	108.89	4979	155.58	124.46
3	4431	138.47	110.77	3923	122.60	98.08
4	6419	**200.59**	160.47	5927	185.21	148.17
5	7677	**239.90**	191.92	5149	160.91	128.73

**Table 10:** Maximum load Fmax [N] and stress σ [MPa] in substrates used in welded-bonded joints (epoxy-based adhesive).

		Al-DC + AB			Al-TL + AB	
Sample No.	Fmax (Al-DC)	σ (DC)	σ (Al)	Fmax (Al-TL)	σ (TL)	σ (Al)
1	9951	**310.99**	248.79	12,082	**377.57**	**302.05**
2	10,368	**324.01**	259.20	12,351	**385.97**	**308.77**
3	10,039	**313.71**	250.96	12,117	**378.65**	**302.92**
4	10,389	**324.65**	259.72	12,716	**397.38**	**317.90**
5	10,336	**323.01**	258.40	12,506	**390.80**	**312.64**

**Table 11:** SEM analysis of cross-sections and fracture surfaces of welded joints.

Joint	Cross-Section of the Joint	Al Sheet Plate after Destruction, Embedded Insert Element	Detail View of Insert Element Embedded in Al Alloy Melt
Al-DC	** 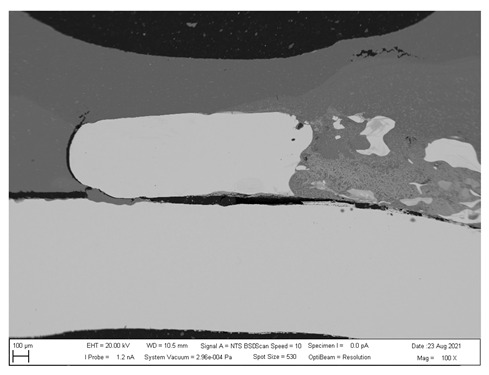 **	** 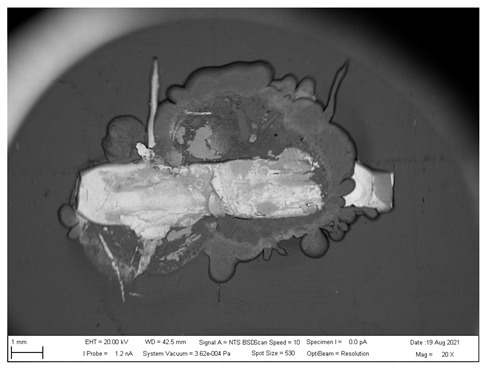 **	** 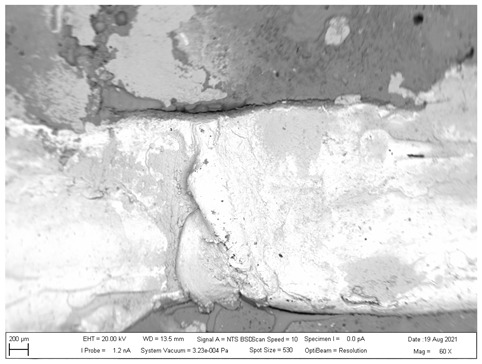 **
Al-TL	** 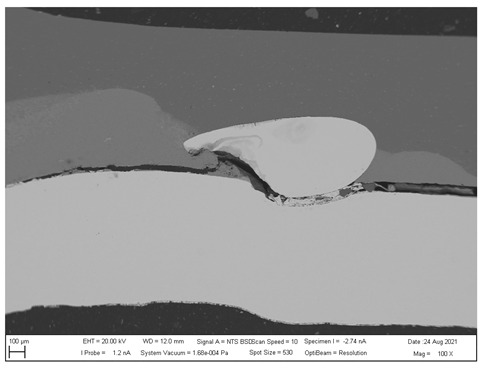 **	** 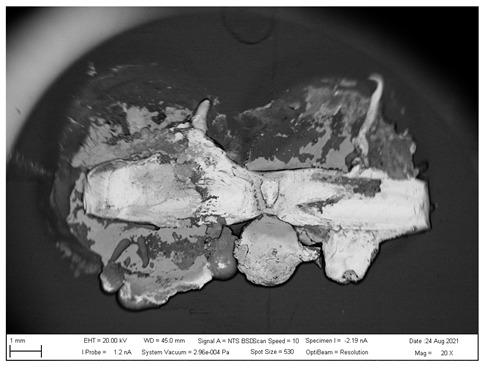 **	** 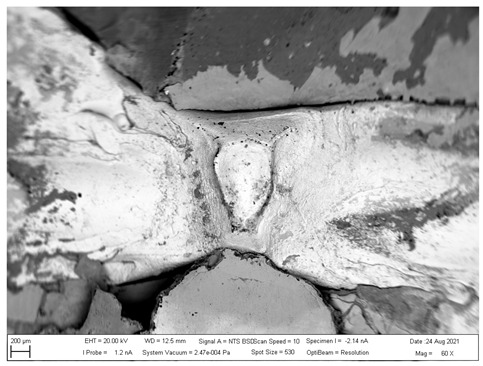 **

## Data Availability

Not applicable.
